# Treatment and outcomes of fractures in children: a registry-based cohort study of 10,144 fractures

**DOI:** 10.2340/17453674.2026.46044

**Published:** 2026-06-12

**Authors:** Topi LAAKSONEN, Petra GRAHN, Matti AHONEN, Juho-Antti AHOLA, Ilkka HELENIUS, Yrjänä NIETOSVAARA

**Affiliations:** 1 Department of Pediatric Orthopedics and Traumatology, New Children’s Hospital, Helsinki University Hospital, Department of Medicine, University of Helsinki, Helsinki; 2 Department of Pediatric Surgery, Kuopio University Hospital, University of Eastern Finland, Kuopio, Finland

## Abstract

**Background and purpose:**

There is no universal consensus on evaluating the quality of pediatric fracture care. While U.S. News ranks hospitals based on surgical timing and procedural benchmarks, additional metrics such as complication rates, reoperations, and patient-reported outcomes could provide a more comprehensive assessment. We aimed to evaluate pediatric fracture care outcomes at a Level I trauma center, emphasizing standardization and transparency in benchmarking.

**Methods:**

This retrospective study reviewed all fractures diagnosed at HUS New Children’s Hospital, Helsinki from 2018 to 2022. U.S. News criteria were applied to completely dorsally displaced supracondylar humerus (Gartland III) fractures, femoral shaft fractures, and displaced forearm fractures to evaluate timeliness, method of treatment, and anesthesia use. The modified Clavien–Dindo–Sink system was used in complex fractures. Permanent iatrogenic nerve injuries and deep infections were registered in all fractures.

**Results:**

10,144 fractures were diagnosed, of which 18% were treated in the operative room. Surgery started within 18 h in 173 (87%) of Gartland III and 108 (79%) of femoral shaft fractures. Open reduction rate was 24% in Gartland III fractures. Most (60%) displaced forearm fractures were managed without general anesthesia. The overall complication rate for Gartland III and femoral shaft fractures was 13%; unplanned return to surgery occurred in 2.2% respectively. Permanent iatrogenic nerve injuries and deep infections were rare (0.04% and 0.3%) in all fractures.

**Conclusion:**

Surgical treatment of supracondylar humerus and femoral shaft fractures in children was performed safely and effectively at our institution. The majority of pediatric forearm fractures were managed in the emergency department without anesthesia. The incidence of iatrogenic nerve injuries and postoperative infections was very low. Incorporating broader metrics such as complications, outcomes, and satisfaction would offer a more complete assessment of care quality.

U.S. News ranks the best children’s hospitals by an online survey, representing the only publicly available annual metric that assesses quality of care in some way. In this ranking, the standard of pediatric orthopedic and trauma services is measured by the ability to prevent surgical complications in scoliosis correction (13 points), the speed and success in treating complex fractures (6 points), and the management of displaced forearm fractures (2 points) [1]. Speed of treatment of completely dorsally displaced supracondylar humerus (Gartland III) and femoral shaft fractures, rate of open reduction in Gartland III fractures, and rate of reduction under anesthesia of displaced forearm fractures are assessed for the ranking [1]. The number of fractures treated, the rate of surgery, experience of the surgeons, complications, and subjective outcomes should also be incorporated into quality evaluation. Regular professional and public reporting using straightforward metrics would enhance transparency and benchmarking. We are not aware of whether any other hospital provides annual open-access reports on pediatric fracture treatment quality.

We aimed to evaluate the quality of pediatric fracture care using the previously mentioned U.S. News criteria over a 5-year period in a Level I pediatric trauma center. Additionally, we assessed the need for immediate reoperation and complications in patients with complicated fractures, as well as iatrogenic nerve injuries, postoperative infections, and patient satisfaction among all surgically treated fracture patients.

## Methods

### Study design

New Children’s Hospital, Helsinki is the largest Level I pediatric hospital in Finland and the only facility providing 24/7/365 on-call pediatric orthopedic services for children aged 0–15 years within a catchment area of 1.5 million people in Southern Finland. A team of 10 attending surgeons (8 orthopedic and 2 hand specialists, each with experience in more than 100 pediatric fracture surgeries) manage all trauma patients, performing surgeries directly or supervising those conducted by registrars either remotely or in the operating room.

Since 2014, all pediatric fractures treated in our hospital have been systematically recorded in the KIDS Fracture Tool, an electronic fracture treatment, quality, and registry device (New Children’s Hospital, Helsinki, Finland and BCB Medical, Turku, Finland). This tool captures comprehensive data, including patient demographics, fracture etiology, anatomical location, fracture-specific details (ICD10 code, closed vs open vs pathological vs re-fracture, Peterson classification of physeal injuries, and treatment method [2], treatment interventions, and complications.

### Outcomes

According to U.S. News’ “Speed and Success in Treating Complex Fractures (page 120)” in “Methodology: U.S. News & World Report, Best Children’s Hospitals 2022–23” by Olmsted et al., timelines of surgery contribute up to 4 points, with Gartland III supracondylar humerus fractures in children aged 0–15 years giving 1 point if 75–90% of patients begin surgery within 18 hand 2 points if ≥ 90% meet this criterion. Similarly, femoral shaft fractures in the same age group give 1 point if 60–80% of surgeries start within 18 h and 2 points if ≥80% are timely. The method of reduction for supracondylar fractures contributes 2 points, awarded if ≤ 5% of procedures are open reductions (2 points) or 5–10% are open reductions (1 point). An additional 2 points are available for managing displaced forearm fractures in children aged 0–13 years, with 1 point given if 70–90% are treated without hospital admission and 2 points if ≥ 90% avoid admission (Table).

**Table:** Proposed core domains for benchmarking pediatric fracture care quality

Description	Measurement	Goal	Current study
**Institutional parameters**			
• Trauma center level	I–IV		
• Fractures treated	n/year		
• Rate of surgical treatment	% of all fractures		
**Core domains**			
• Timeline of treatment			
Gartland III supracondylar humerus fractures	to OR < 18 h **^a^**	≥ 90%	87%
Femoral shaft fractures	to OR < 18 h **^a^**	≥ 90%	79%
• Method of treatment			
Open reduction/Gartland III fractures	%	5–30%	24%
No surgery/displaced forearm shaft fractures	%	50–80%	62%
• Complications of treatment			
Unplanned return to OR all fractures	%	< 1%	NA
Permanent nerve injury from surgery	%	0%	0.04%
Rate of complications all fractures **^b^**	Clavien–Dindo–Sink (3–5)	0%	NA
Deep SSI after surgical treatment of closed fractures **^b^**	%	< 1%	0.3%
• Service user satisfaction			
Patient	(0–10) **^c^**	> 90% ≥ 8	
Guardian	(0–10) **^c^**	> 90% ≥ 8	92%

a Time to operating room (OR) from admission in hours

b Optional assessment of treatment complications using the Clavien–Dindo–Sink classification and surgical site infection (SSI) rates.

c 0 (poor) – 10 (excellent)

NA: not applicable.

All children under 16 years of age who were given a fracture diagnosis in New Children’s Hospital, Helsinki over a 5-year period (2018–2022) were included. The number of children treated under general anesthesia in the operating room (OR) was recorded.

Following the “Speed and Success in Treating Complex Fractures” standards [1], we identified all children aged 0–15 years with grade III supracondylar humerus and femoral shaft fractures, as well as children aged 0–13 years with displaced forearm fractures. For patients with completely dorsally displaced supracondylar humerus Gartland III or femoral shaft fractures, the time from hospital admission to the start of surgery was recorded, and the percentage of surgeries initiated within 18 h of admission for each group was calculated. We also assessed the institutional and surgeon specific rate of open reduction in treating Gartand III fractures. Additionally, the percentage of displaced distal or diaphyseal forearm fractures (ICD10 S52.2–6) managed without general anesthesia or hospital admission was calculated.

The risk of unplanned returns to the OR after Gartand III or femoral shaft fracture surgery was assessed. Also, the risks for postoperative infections and iatrogenic nerve injuries were recorded for all fracture surgeries. To evaluate the overall complication rate in Gartland III or femoral shaft fractures the modified Clavien–Dindo–Sink (CDS) classification system was utilized [3]. Modified CDS categorizes complications based on the level of intervention required, ranging from minor deviations in standard care that require no intervention (Grade I) to life-threatening complications or death (Grades IV and V). Designed to standardize the evaluation of complications after surgical treatment, the CDS system facilitates consistency in reporting and comparison across procedures and institutions [3,4].

Additionally, since 2021, guardian satisfaction with surgical treatment has been assessed using the “MyHealth” questionnaire distributed through the KIDS Fracture Tool. Guardians’ overall satisfaction with their child’s fracture care has been assessed by 10-point Likert scale (1 = very dissatisfied, 10 = very satisfied) at 2, 8, and 26 weeks after surgery since 2021.

To enhance transparency and support benchmarking, the aforementioned data has been published on the hospital’s website (www.hus.fi/en/professionals/our-treatment-results/treatment-results-new-childrens-hospital/treatment-results) after finalizing the study. The STROBE guidelines were followed.

### Statistics

Statistics data is presented descriptively as numbers and percentages with means and range as appropriate.

### Ethics, data sharing plan, funding, use of AI, and disclosures

The study protocol was approved by the Helsinki University Hospital Review Board (365/13/03/03/2015). Data is available from the corresponding author. No artificial intelligence tools were used in the conduct or writing of this study. This study was supported by research grants from the Finnish Paediatric Research Foundation. Complete disclosure of interest forms according to ICMJE are available on the article page, doi: 10.2340/17453674.2026.46044

## Results

**Figure 1:**
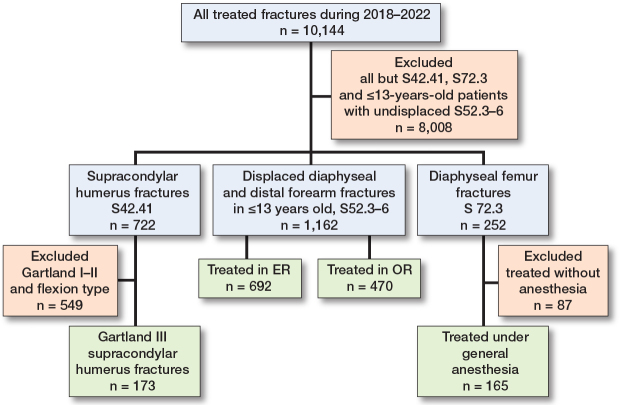
Flowchart including all fractures in children less than 16 years old treated in New Children’s Hospital, Helsinki during the 5-year study period. ER: emergency room, OR: operating room

10,144 fractures were treated in under-16-year-old children during the 5-year study period (mean number of fractures annually 2029, range 1,517–2,255) (Figure 1). The majority (n = 6,421, 63%) of all fractures were treated without reduction with immobilization and 1,937 (19%) with immobilization after manipulation in local anesthesia. The ratio of fractures treated under general anesthesia varied annually between 1,618 (16%) and 2,110 (21%), mean 1,806 (18%). All 173 Gartland III fractures and 165 (66%) femoral shaft fractures were treated under general anesthesia; these accounted for 9.7% and 6.1%, respectively, of all 2,170 fractures treated under general anesthesia.

### Time to surgery with complex fractures

**Figure 2:**
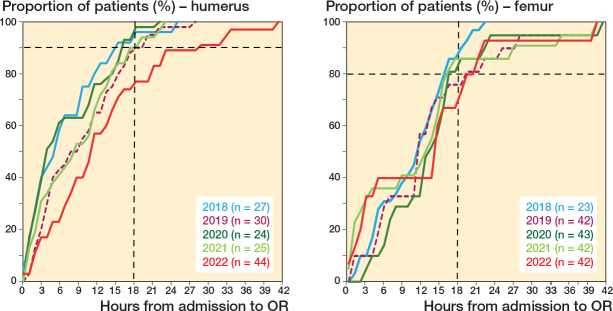
Time from admission to the operating room for 173 children with completely displaced supracondylar humerus fractures (left panel) and for 108 children with femoral shaft fractures (right panel) treated in the New Children’s Hospital, Helsinki, Finland, during 2018–2022.

The mean time from admission to OR in Gartland III fractures was 10 h (median 8, range 0–43 h), with 87% admitted to the OR within 18 h (Figure 2). The mean time from admission to OR for children with femoral shaft fractures was 13 h (range 0–43 h) and 79% reached the OR within 18 h (Figure 2).

Of the 1,162 displaced distal or diaphyseal forearm fractures in patients 13 years or younger 692 (60%) were treated using local anesthesia and/or conscious sedation in the emergency room (ER). Of the 794 displaced forearm shaft fractures in children below 16 years of age, 62% were treated nonoperatively.

### Surgeon-specific volume and reoperations

The mean number of Gartland III fracture operations per attending surgeon was 14 (range 3–22) during the 5-year study period. Registrars performed 29 (17%) of the operations without attending-surgeon supervision in theatre. The overall rate of open reduction of Gartland III fractures in our institution was 24%, the surgeon-specific rate varying between 0% and 67%.

3 children (1.7%) with Gartland III fractures treated with lateral Kirschner wires required reoperation due to loss of reduction within a week (n = 2) or malunion (n = 1). 3 children (3.5%) with femoral shaft fractures had reoperation 6–41 days from primary surgery: too short plate (n = 1), and wrong size flexible intramedullary nails (n = 2).

### Complications

Across the entire cohort of surgically treated children, 22 (< 1%) superficial infections and 7 (0.3%) deep postoperative infections were identified. Half of the superficial infections occurred among the altogether 302 children who underwent pin fixation of a Gartland II, III, and flexion type of supracondylar humerus fracture. Iatrogenic nerve injuries were recorded in 20 (19 transient, 1 permanent) surgically treated patients. Half of the injuries (n = 10) were caused during pin fixation of supracondylar humerus fractures, half during flexible intramedullary nailing of forearm shaft fractures including the 1 permanent injury (sensory branch of the radial nerve).

The mean overall complication rate according to the CDS classification for management of Gartland III and femoral shaft fractures during the study period was 13%: Grade I 5.4%, II 5.7%, and III 1.8%. Of the CDS grade III complications 5 were registered in children with Gartland III fractures (2 reoperations, 2 deep infections, and 1 malunion).

### Satisfaction

The mean guardian’s (n = 152/1,044, 15%) overall satisfaction according to the My-Health questionnaire on surgical treatment was 9.2 (range 2–10).

## Discussion

We aimed to evaluate how quality in pediatric fracture care can be assessed using institutional outcome reporting and currently available benchmarking criteria. We showed that although our institution met most U.S. News benchmarks for timeliness of care, broader measures such as complications, reoperations, and patient- and guardian-reported outcomes may better reflect the true quality of pediatric fracture care.

Quality of care is a critical component of modern healthcare, particularly as efficiency, transparency, and cost-effectiveness gain increasing importance [4,5]. Despite its emphasis on surgical timing and avoidance of complications in scoliosis surgery, the U.S. News framework for ranking pediatric hospitals does not account for metrics such as complication rates, reoperations, or patient-reported outcomes in pediatric fracture care. Our study demonstrates that these additional measures, alongside standard benchmarks, provide a more comprehensive evaluation of care quality, as suggested by Gelfer et al. [6].

In our study, surgery for Gartland III supracondylar humerus fractures commenced within 18 h in 151 (87%) cases and in 130 (79%) for femoral shaft fractures. While these results align well with U.S. News benchmarks, existing evidence challenges the centrality of surgical timing as a quality indicator. Studies by Grauberger et al. and Ondina et al. found no significant association between delays and adverse outcomes in femoral shaft and supracondylar fractures, respectively [7,8]. Instead, fracture complexity, neurovascular compromise, and severe displacement are stronger predictors of surgical outcomes [9]. Strict adherence to timeliness metrics risks prioritizing speed over optimizing surgical conditions, such as daytime operations with experienced surgeons [10-13], although speed of treatment is undoubtedly important for patients and caregivers.

Our open reduction rate for Gartland III fractures (24%) exceed the U.S. News threshold of ≤ 5% yet aligns with published rates of 7–30% [14,15]. Similarly, only 679 (60%) displaced forearm fractures in children aged 13 years or younger were treated without general anesthesia in the emergency room, falling short of the U.S. News benchmark of ≥ 70%. This could be attributed to a change of injury patterns due to high-energy fractures from, e.g., trampoline and e-scooter accidents [16-19]. Rigid benchmarks focused on procedural avoidance may penalize institutions managing more severe injuries, highlighting the need to account for fracture complexity. Furthermore, options belong to modern care and fracture reduction under anesthesia should be offered for overly anxious children and their guardians.

Complication rates, reoperations, and patient-reported outcomes are critical indicators of care quality yet remain absent from existing pediatric fracture benchmarks. Transparent reporting of these metrics, as demonstrated by publishing our results online, supports healthcare systems in identifying inefficiencies and improving resource allocation [4,5]. For example, our low rates of unplanned returns to the OR (n = 6, 2.2%) and deep infections (n = 2, 0.3%) compare favorably with published data and underscore the value of standardized reporting [14,20]. Incorporating these metrics into benchmarking frameworks would provide a more accurate reflection of care quality and promote cost-effective practices.

Our findings highlight the limitations of the U.S. News criteria, particularly their narrow focus on surgical timing and procedural avoidance. Critically, these criteria appear to measure hospital resources more than the actual quality of care delivered. Results are reported as composite scores, meaning that detailed outcome data is not available to enable meaningful comparisons of true treatment outcomes [1]. In the Nordic countries, healthcare systems are predominantly publicly funded. As a result, superior outcomes achieved at one hospital do not directly impact others, since there is no competition for patients or associated revenue. Rather, better outcomes at one institution should serve as an example and an incentive for others to improve care quality and allocate resources more effectively [5–6].

In our view, complication rates, reoperation, and patient- or guardian-reported outcomes are key indicators of quality in pediatric fracture care and should be incorporated into ranking systems (Table). In the future, children and their parents should also be asked what they consider to be high-quality fracture care [21]. To our knowledge, no other institutions currently publish pediatric fracture outcomes on a regular and transparent basis.

### Strengths

The large cohort of 10,144 pediatric fracture patients diagnosed over 5 years provides robust data for assessing care quality. The use of standardized tools, such as the modified Clavien–Dindo–Sink classification and the MyHealth questionnaire, ensures consistency and comparability in outcome measurement. Publishing these results online promotes transparency and represents a step toward open benchmarking.

### Limitations

The results represent the experience of a single tertiary pediatric trauma center and may not be directly generalizable to all healthcare systems or institutions. Although the fracture registry was prospectively maintained, the present evaluation was retrospective in nature. In addition, patient- and guardian-reported outcome collection was introduced during the study period, resulting in a relatively low response rate (15%).

However, these limitations also reflect the current challenges in benchmarking pediatric fracture care internationally. The lack of standardized reporting systems and comparable external datasets highlights the need for broader collaboration and more uniform quality assessment methods. 

### Conclusion

Surgical treatment of supracondylar humerus and femoral shaft fractures in children was performed safely and effectively at our institution. The majority of pediatric forearm fractures were managed in the emergency department without anesthesia. The incidence of iatrogenic nerve injuries and postoperative infections was very low.

Broader outcome measures—including complications, reoperations, and patient- and guardian satisfaction—offer a more comprehensive and clinically meaningful evaluation. Reporting of outcomes that truly reflect the quality of care would improve transparency, enable more meaningful comparisons between institutions, and support the development of high-quality, cost-effective pediatric fracture care.

## Notes

TL: Conceptualization, Methodology, Data curation, Visualization, Writing – original draft. PG: Validation, Methodology, Writing – original draft. MA: Methodology, Validation, Writing – review & editing. J-AA: Data curation, Writing – review & editing. IH: Conceptualization, Methodology, Writing – review & editing, Funding acquisition. YN: Conceptualization, Methodology, Supervision, Writing – review & editing

The authors would like to express their sincere thanks to reviewer Sebastian Strøm Rönnquist for the exceptionally helpful comments and constructive suggestions, which significantly improved this manuscript.

Handling co-editors: Ivan Hvid and Robin Christensen

*Acta* thanks Klaus Dieter Parsch and Sebastian Strøm Rönnquist for help with peer review of this manuscript.
